# Targeting insulin-like growth factor pathways

**DOI:** 10.1038/sj.bjc.6602963

**Published:** 2006-01-31

**Authors:** D Yee

**Affiliations:** 1 University of Minnesota Cancer Center, Department of Medicine, MMC 806, 420 Delaware Street SE, Minneapolis, MN 55455, USA

**Keywords:** insulin-like growth factors, insulin-like growth factor receptors, monoclonal antibodies, tyrosine kinase inhibitors

## Abstract

Some cancer cells depend on the function of specific molecules for their growth, survival, and metastatic potential. Targeting of these critical molecules has arguably been the best therapy for cancer as demonstrated by the success of tamoxifen and trastuzumab in breast cancer. This review will evaluate the type I IGF receptor (IGF-IR) as a potential target for cancer therapy. As new drugs come forward targeting this receptor system, several issues will need to be addressed in the early clinical trials using these agents.

There are several excellent reviews outlining a rationale for targeting the IGF system in cancer (for example Pollak *et al*, 2004). Indeed, the first clinical trials testing a monoclonal antibody directed against the type I IGF receptor (IGF-IR) are currently underway. Thus, the questions have turned away from ‘should we target this pathway?’ to several other questions concerning best clinical strategies for anti-IGF therapies. This mini-review will address questions to address in the upcoming clinical trials.

## WHAT ARE THE TARGETS?

Like many other growth factor systems, the IGF system consists of more than a single ligand interacting with a single receptor. There are three ligands (IGF-I, IGF-II and insulin) that interact with at least four receptors. In addition, the IGF system also involves six well characterized binding proteins that regulate IGF action (Clemmons, 1998). IGF-IR is a transmembrane tyrosine kinase that is highly related to insulin receptor (IR). The function for IR in health and disease is well known. IGF-IR plays an important role in childhood growth as demonstrated in both humans and mice (Liu *et al*, 1993; Abuzzahab *et al*, 2003). In addition to these holoreceptors, hybrids between IGF-IR and IR have exist (Federici *et al*, 1997). Interestingly, two splice variants of IR have been documented. The IR-A fetal splice variant is more frequently found in cancer (Frasca *et al*, 1999). The type II IGF receptor (IGF-IIR) has high affinity for IGF-II, but does not apparently transmit an extracellular signal. This receptor has been characterized as a ‘sink’ for IGF-II and its loss has been demonstrated in human cancer (MacDonald *et al*, 1988). Thus, in the extracelluar space, the IGF ligands have potential interactions with four receptors and six binding proteins.

IGF-IR, IR, and hybrid receptors all function as covalently linked dimers. As such, physiologic activation of these receptors by overexpression alone is not seen, in contrast to the epidermal growth factor (EGF) receptor family members. While there are some cell model studies demonstrating activation of IGF-IR by overexpression of receptor or intracellular tyrosine kinases (Kozma and Weber, 1990), activation of IGF-IR requires binding to ligand in most physiologic settings. This important fact has implications for anti-IGF therapy as noted below.

To date, much of the focus has been placed on IGF-IR. However, it must be remembered that IR may play a role in cancer cell biology. At physiologic concentrations of insulin, breast cancer cells are stimulated to proliferate *in vitro* (Osborne *et al*, 1976). In addition, activation of IR-A by IGF-II has been demonstrated in breast cancer cell lines (Sciacca *et al*, 1999). Thus, inhibition of both IGF-IR and IR may be required for optimal suppression of IGF signalling pathways. This possibility adds a layer of complexity to targeting the IGF system, as dysfunctional IR signalling is well understood and results in type II diabetes.

These preclinical data suggest that targeting of only IGF-IR may be insufficient to block tumor growth regulated by the IGFs and insulin. Since inhibition of IR could have profound effects on host glucose homeostasis, further definition of IR as an important target in cancer cells is needed.

## WHAT IS THE BEST STRATEGY TO BLOCK IGF ACTION?

Given the requirement for ligand activation of IGF-IR signalling, one possible strategy to block this pathway would be to lower IGF-I concentrations. During puberty, growth hormone (GH) release from the pituitary gland results in the production of IGF-I by the liver. Thus, disruption of the hypothalamic–pituitary–liver axis could result in lowered serum IGF-I levels. Several GH releasing hormone (GHRH) antagonists have been described and have antitumour activity in cancer model systems (Letsch *et al*, 2003). A pegylated mutant GH (pegvisomant) has been developed to disrupt GH signalling in patients with acromegaly, a condition of GH excess. This compound also has antitumour activity (McCutcheon *et al*, 2001). While the precise mechanisms for the antitumour activity of these compounds is not completely understood, as both GHRH and GH antagonists may have direct antitumour activity, they both result in suppression of serum IGF-I levels.

While these drugs could have activity, they do not affect IGF-II levels. Since mice and rodents do not express IGF-II during adulthood (DeChiara *et al*, 1991), it has been difficult to model IGF-II action *in vivo*. Neutralization of IGF ligands could also be effective therapeutic strategies. Pharmacologic antagonism of IGF ligands has been accomplished by neutralizing monoclonal antibodies and IGF-binding proteins (Van den Berg *et al*, 1997; Goya *et al*, 2004). Since these agents would only target the IGF ligands, insulin action would be relatively unaffected.

The bulk of drug development has been directed towards targeting IGF-IR. Several monoclonal antibodies directed against IGF-IR have been created (Li *et al*, 2000; Burtrum *et al*, 2003; Maloney *et al*, 2003; Goetsch *et al*, 2005; Wang *et al*, 2005). To date, all antibodies seem to have a similar mechanism of action and result in IGF-IR downregulation. Unlike binding of the native ligands that allow receptor recycling, several laboratories have shown that monoclonal antibody binding results in endosomal degradation of the receptor (Sachdev *et al*, 2003; Wang *et al*, 2005). While most of the antibodies also block ligand binding and inhibit the activation of the receptor, it is notable that a fully agonistic antibody also has antitumour properties (Li *et al*, 2000; Sachdev *et al*, 2003). This is presumably due to the antibody's ability to downregulate receptor expression. Thus, the agonist properties of the antibody may be less relevant than the ability to downregulate receptor levels over time.

Since IGF-IR is a tyrosine kinase, small molecule inhibitors have also been developed (Garcia-Echeverria *et al*, 2004; Mitsiades *et al*, 2004; Carboni *et al*, 2005). While these compounds show some selectivity of IGF-IR over IR in cell model systems, it is uncertain as to whether this selectivity can be seen *in vivo*. Moreover, whether or not IGF-IR selectivity is even desirable is an open question. As noted above, if IR mediates a substantial portion of IGF-stimulated tumour cell biology, especially via IGF-II activation, then cancer cell inhibition of IR would be necessary.

In addition to small molecule inhibitors, which mostly bind the catalytic site of IGF-IR, other compounds have been described that disrupt receptor function. A cyclolignan, picropodophyllin, has been shown to disrupt IGF-1R activation by interfering with substrate phosphorylation (Vasilcanu *et al*, 2004). This compound appears specific for IGF-IR and has been shown to have activity in several model systems (Girnita *et al*, 2004; Stromberg *et al*, 2006). As noted above, complete selectivity for IGF-IR would have obvious benefits for host glucose may be insufficient for complete disruption of tumour signalling. Another compound, nordihydroguaiaretic acid (NDGA) has also been shown to disrupt IGF-IR activation (Youngren *et al*, 2005). Compared to picropodophyllin, this NDGA is not specific for IGF-IR but also blocks human epidermal growth factor-2 (HER2) signalling. While this might seem undesirable, recent data suggest that IGF-IR plays a role in resistance to the anti-HER2 monoclonal antibody trastuzumab (Nahta *et al*, 2005). Thus, a drug that disrupts both pathways may be of value in specific situations.

Carefully designed clinical trials will be necessary to examine the effect on host glucose metabolism. While temporary inhibition of IR function and resultant hyperinsulinemia or hyperglycemia could be tolerated, insulin resistance over long periods of time would surely be detrimental to patients. Moreover, there are animal model systems showing that disruption of IGF-IR affects survival of the pancreatic beta cells (Withers *et al*, 1999). In a worst case scenario, long-term inhibition of IGF-IR could induce type I and type II diabetes by inhibiting insulin secretion and insulin action, respectively.

## ARE THERE BIOMARKERS FOR IGF ACTION?

The development of trastuzumab and the EGFR tyrosine kinase inhibitors have demonstrated that careful measurement of biomarkers is necessary when only a minority of patients have ‘receptor driven’ tumours. In contrast, tamoxifen was initially given to breast cancer patients unselected for estrogen receptor-*α* (ER*α*) status. Eventually it became clear that only ER*α* expressing patients responded to tamoxifen. However, the early unselected clinical trials were successful because ER*α* is expressed by the majority of tumours and a high percentage of those ER*α* positive tumours respond to tamoxifen as a single agent. Thus, biomarkers do not need to be defined if a substantial number of tumours express the target and inhibition of the target causes a clinical response in a high proportion of target-bearing tumours.

While it is clear that IGFs stimulate growth of many tissues, it is difficult to show evidence of IGF-IR activation, even in the most favourable settings. In animal models of IGF action, administration of exogenous IGF is necessary to show activation of IGF signalling pathways (Pete *et al*, 1999). At a minimum, IGF-IR must be present, but beyond this requirement, the ‘signature’ of an IGF-driven tumour is unclear.

Measurement of key signalling pathways immediately downstream of IGF-IR offers some insight into IGF action. In model systems, the expression of insulin receptor substrate (IRS) molecules is necessary to couple IGF-IR with key downstream signalling pathways. In breast cancer cells, specific IRS adaptor proteins are activated downstream of IGF-IR that link the receptor to specific phenotypes. For example, IRS-1 activation is associated with IGF-stimulated proliferation, while IRS-2 signalling is necessary for metastatic behaviour (Jackson *et al*, 1998, 2001; Nagle *et al*, 2004; Zhang *et al*, 2004). While phosphorylation of IRS proteins has been difficult to show in primary human breast cancers, it is possible that expression of both IGF-IR and distinct IRS species could be used to identify cancers most likely to respond to IGF-IR inhibition.

## HOW SHOULD IGF INHIBITORS BE TESTED IN COMBINATION WITH OTHER DRUGS?

The combination of targeted agents with conventional cytotoxic drugs has provided important insight into the therapeutic synergy. Disruption of HER2 or EGFR enhances cytotoxic drug activity in some, but not all, cancers. Perhaps the most striking example of synergy occurs with the coadministration of trastuzumab with the taxanes. First demonstrated in metastatic breast cancer (Slamon *et al*, 2001), the synergy is even more dramatic in the adjuvant therapy of breast cancer (Piccart-Gebhart *et al*, 2005; Romond *et al*, 2005). Similarly, cetuximab in combination with irinotecan demonstrates therapeutic benefit, even when tumours have progressed beyond irinotecan alone (Cunningham *et al*, 2004). In contrast, gefitinib in combination with paclitaxel and carboplatinum in nonsmall cell lung cancer failed to show any evidence for therapeutic synergy (Herbst *et al*, 2004). Furthermore, tamoxifen given simultaneously with chemotherapy in the adjuvant treatment of breast cancer reduces the benefit for chemotherapy (Albain *et al*, 2002).

These trials show that the synergy between targeted therapies and conventional chemotherapy is not necessarily easily predicted. On one hand, the targeted therapy may induce apoptosis and lower the survival threshold of cancer cells thereby augmenting a second apoptotic stimulus. Trastuzumab, when given as a single agent, induced apoptosis in primary breast cancers (Mohsin *et al*, 2005) and this could be the mechanism of action for the observed benefit of combined therapy. In contrast, some targeted therapies, such as tamoxifen, antagonize the effects of chemotherapy, potentially by altering progression through the cell cycle or by affecting transport of drugs (Osborne *et al*, 1989).

Inhibition of IGF-IR may act in either fashion and may be dependent on the strategy used to block IGF signalling. Monoclonal antibodies directed against IGF-IR induce tumour cell apoptosis in preclinical model systems and have been shown to synergize with chemotherapy. It has been suggested that downregulation of IGF-IR induces apoptotic cell death (Baserga, 2005). Since all of the currently described antibodies have demonstrated the ability to downregulate receptor, this may be an important mechanism of synergy. In contrast, tyrosine kinase inhibitors successfully inhibit the biochemical activity of IGF-IR and do not appear to downregulate receptor levels. Thus, the tyrosine kinase inhibitors may block progression through S-phase without inducing apoptosis. If this is the case, then the kinase inhibitors may actually interfere with the cell cycle specific effects of chemotherapy similar to the interference observed between tamoxifen and cytotoxic treatment. Careful evaluation of synergy between IGF-IR disruption and combination therapy must be studied in combination clinical trials.

## WHAT'S WRONG WITH IGF ACTION AS A TARGET?

Anti-IGF-IR trials will first be tested in patients with advanced cancers. As noted above, identifying patients with IGF-driven tumours may be difficult. In addition, IGF-IR has many effects on cancer cells that are not easily measured in a phase II clinical trial. For example, IGF-IR activation stimulates motility in many cancer cells. In some cells, IGF-IR activation does not apparently effect proliferation or survival. Thus, these types of cells have fully intact IGF-IR signalling pathways yet lack a phenotypic response to IGF inhibition that can be easily measured in clinical trials. Even the most carefully designed studies with appropriate and robust biomarker measurement would be unable to identify this type of IGF effect.

IGF-IR is ubiquitously expressed in most normal tissues (Werner *et al*, 1991). In this regard, targeting IGF-IR is different than targeting estrogen receptor where relatively limited expression of the receptor is seen. For example, IGF-IR enhances neuronal survival, maintains cardiac function, stimulates bone formation and hematopoiesis (Zumkeller, 2002; Rosen, 2004; Leinninger and Feldman, 2005; Saetrum Opgaard and Wang, 2005). IGF-IR signalling has also been shown to play a role in maintaining survival of pancreatic beta cells (Withers *et al*, 1999). Thus, disruption of IGF-IR could result in many potential toxicities. Of course, these concerns could be raised about essentially every successful cancer therapy. Inhibition of basic cellular processes, such as nucleotide synthesis, tubulin function, and DNA replication have all been proven to be of value in the treatment of cancer despite many potential toxicities. The ongoing clinical trials will determine whether long- or short-term inhibition of IGF-IR results in unacceptable toxicity. Hopefully, these studies will define a therapeutic window for IGF-IR inhibition.

## CONCLUSION

There are many reasons to consider IGF-IR as a target for cancer prevention and therapy. Similarly, there are also many potential methods to disrupt IGF-IR signalling. Finally, there are numerous potential toxicities associated with disruption of IGF-IR activation in normal tissues. As clinical trials move forward, we will determine what is ‘potential’, what is of clinical relevance, and whether disruption of this growth factor pathway results in relevant clinical outcomes.
